# Comprehensive evaluation of nucleic acid amplification methods widely used for generic detection of sandfly-borne phleboviruses

**DOI:** 10.1128/spectrum.03428-23

**Published:** 2024-03-08

**Authors:** Ceylan Polat, Nazli Ayhan, Koray Ergünay, Remi N. Charrel

**Affiliations:** 1 Department of Medical Microbiology, Faculty of Medicine, Hacettepe University, Ankara, Turkey; 2 Unité des Virus Emergents, Aix Marseille University, Marseille, France; 3 National Reference Center for Arboviruses, National Institute of Health, and Medical Research (Inserm) and French Armed Forces Biomedical Research Institute (IRBA), Marseille, France; 4 Walter Reed Biosystematics Unit (WRBU), Smithsonian Institution Museum Support Center, Suitland, Maryland, USA; 5 One Health Branch, Walter Reed Army Institute of Research (WRAIR), Silver Spring, Maryland, USA; 6 Department of Entomology, Smithsonian Institution-National Museum of Natural History (NMNH), Washington, DC, USA; 7 Laboratoire des Infections Virales Aigues et Tropicales, Pole des Maladies Infectieuses, AP-HM Hopitaux Universitaires de Marseille, Marseille, France; Universidade Federal do Rio de Janeiro, Brazil

**Keywords:** phlebovirus, sand fly, arbovirus, RT-PCR assay, *in silico*

## Abstract

Sandfly-borne phleboviruses (SBPs), which cause sandfly fever, aseptic meningitis, encephalitis, and meningoencephalitis, are emerging pathogens of major public health concern. Virus nucleic acid testing is essential for SBP diagnosis, especially in the early stages of infection, and for the discovery of novel SBPs. The efficacy of utilizing generic primers that target conserved nucleotide sequences for the detection of both known and novel SBPs has not been extensively evaluated. We aimed to compare and evaluate the performance of five generic primer sets, widely used to detect S- and L-segments of arthropod-borne phleboviruses and designed as singleplex (*n* = 3) and nested (*n* = 2) formats, including both well-known and recently characterized 15 Old World virus strains. Furthermore, we performed *in silico* analysis to assess the detection capabilities of these generic primer sets. The initial evaluation of previously published generic primer sets for SBP detection yielded two singleplex primer sets with the potential to be adapted for use in real-time or high-throughput detection settings. Studies are ongoing to develop and further optimize a preliminary assay and test various hosts and vectors to assess their capacity to detect known and novel viruses.

**IMPORTANCE:**

Virus nucleic acid testing is the primary diagnostic method, particularly in the early stages of illness. Virus-specific or syndromic tests are widely used for this purpose. The use of generic primers has had a considerable impact on the discovery, identification, and detection of Old World sandfly-borne phleboviruses (OWSBP). The study is significant because it is the first to carry out a comparative evaluation of all published OWSBP generic primer sets.

## INTRODUCTION

The genus *Phlebovirus* (order *Bunyavirales*, family *Phenuiviridae*) includes several viruses with significant global public health impacts (1). Similar to other families of the *Bunyavirales* order, infectious phlebovirus particles encapsidate their negative-sense single-stranded RNA genomes in three parts, comprising small (S), medium (M), and large (L) segments, encoding for the virus nucleoprotein, glycoproteins, and replicase, respectively (2). The best-known pathogenic phleboviruses are mainly vector-borne, with sandflies acting as arthropod vectors involved in the transmission to susceptible vertebrates. Sandfly is a colloquial name for flying, biting, and blood-sucking dipterans of the subfamily *Phlebotominae* within the *Psychodidae*. Within the *Phenuiviridae* family, phleboviruses isolated from and vectored by sandflies are functionally regarded as a distinct group, frequently called sandfly-borne phleboviruses (SBPs) (2). SBPs are widely distributed according to the geographical range of their vector species, which include *Phlebotomus* and *Sergentomyia* sandflies in the Old World sandfly-borne phleboviruses (OWSBPs) and *Lutzomyia* sandflies in the New World (3). In Eurasia, SBPs are distributed across various regions, including the Mediterranean Basin, the Middle East, Central Asia, Africa, and the Indian subcontinent.

Human infections due to OWSBPs frequently remain asymptomatic, as observed in many arthropod-borne virus infections of endemicity. Well-known clinical presentations due to OWSBPs include “sandfly fever,” typically produced by sandfly fever Naples virus (SFNV) and sandfly fever Sicilian virus (SFSV), and characterized by self-limiting fever, headache, malaise, photophobia, abdominal symptoms, and skin rash (4). Toscana virus (TOSV) stands out among OWSBPs due to its neurotropism, resulting in central and peripheral nervous system involvement in a subset of infected individuals, presenting as aseptic meningitis, encephalitis, and meningoencephalitis, as well as several peripheral neurological manifestations (5). TOSV is a prominent neurovirulent virus in the Mediterranean Basin, with several cases annually documented during sandfly active seasons (4). Overall, many OWSBPs are considered emerging and re-emerging pathogens that pose significant public health concerns. In many countries, symptomatic infections with multiple OWSBPs are documented, with the co-circulation of several strains in sandflies (6–13).

Virus nucleic acid testing (NAT) is the mainstay diagnostic approach, especially during the early stages of infections prior to the adaptive immune response and specific antibody production in the host. For this purpose, virus-specific or syndromic assays are commonly performed, targeting the most frequently observed pathogens, such as TOSV or SFSV (14–17).

There is a broad genetic diversity among OWSBPs, manifesting as nucleotide variations located in open reading frames or non-translated regions of individual virus genome segments (4). In addition, several novel OWSBPs have been characterized during the last decades, mainly during vector surveillance activities in countries with endemic circulation (1, 6, 7, 18–27). For vector screening, NAT incorporating generic or pan-phlebovirus primers has been a common approach, at times employed to support diagnostic testing as well (28). Targeting genus- or species-based conserved nucleotide patterns on viral replicase or nucleocapsid, these primers are capable of amplifying the majority of the known OWSPBs, as well as detecting novel strains due to the preserved functional motifs selected for primer design (29–33). The initial detection of particular novel OWSBPs was accomplished by using NATs with generic primers, which subsequently led to virus isolation attempts and complete genome sequencing. There are also examples of partial sequences suggesting a novel OWSBP, without successful virus isolation (19). Hence, the use of such generic primers has been significantly impactful on OWSBP discovery, identification, and detection.

Several NATs with generic primers capable of amplifying partial L and S segments of phleboviruses in singleplex and nested PCR formats have been described (29–33). Despite their wide detection range and frequent use, scarce information is currently available on the performance of these sets. In this study, we aimed to carry out a comparative evaluation of all published OWSBP generic primer sets, using a standardized NAT approach and several pathogenic and non-pathogenic virus strains, including well-known as well as recently characterized isolates.

The aim of this study was to assess the performance of five *Phlebovirus* generic assays for detecting OWSBPs using selected viral strains representative of the genetic diversity combined with *in silico* analysis.

## MATERIALS AND METHODS

### Viruses and protocols

A total of 15 SBPs representing 12 virus species circulating exclusively in sand flies from the OWSBPs were included in the study (Table 1). Well-characterized viral strains were obtained from the European Virus Archive Global web catalog (https://www.european-virus-archive.com/evag-portal/) and were used as provided without further cell culture propagation (GenBank accession numbers are indicated in Table 1). The 15 strains consisted of OWSBPs (i) causing human infections such as TOSV lineages A and B, SFNV and SFSV, and (ii) with possible or unexplored human pathogenicity (Table 1).

**TABLE 1:** Virus strains used for generic SBP amplification

Serocomplex	Species	Virus (acronym)	TCID50/mL	Strain ID	Host	Sequence accession	
Salehabad	*Adana phlebovirus*	Adana virus (ADAV)	10E7.82	195	*Phlebotomus* sp.	NC_029127 (L)	NC_029129 (S)
Sicilian	*Corfou phlebovirus*	Corfou virus (CFUV)	10E3.82	PaAr 814	*Ph. major*	KR106177 (L)	KR106179 (S)
Naples	*Massilia phlebovirus*	Massilia virus (MASV)	10E7.22	FR M43	*Ph. perniciosus*		
Salehabad	*Medjerda Valley phlebovirus*	Medjerda Valley virus (MVV)	10E7.42	T131	*Phlebotomus* sp.	NC_055383 (L)	NC_055381 (S)
Naples	*Naples phlebovirus*	SFNV	10E4.57	IT 30451	*Phlebotomus* sp.	GQ165528 (L)	
Naples	*Punique phlebovirus*	Punique virus (PUNV)	10E7.82	TN T101	*Phlebotomus* sp.	OM362898 (L)	
Salehabad	*Salehabad phlebovirus*	Arbia virus (ARBV)	10E6.42	Phl.35 M6	*Phlebotomus* sp.	EU266620 (L)	GQ165524 (S)
Salehabad	*Salehabad phlebovirus*	Zaba virus (ZABAV)	10E6.57	HR/C48	Ph. neglectus	MG573142 (L)	MG573147 (S)
Salehabad	*Salehabad phlebovirus*	Salehabad virus (SALV)	10E7.42	I-81	*Phlebotomus* sp.	JX472403 (L)	JX472405 (S)
Sicilian	*Sicilian phlebovirus*	SFSV	10E6.82	Sabin	*Homo sapiens*	EF095551 (L)	EF201822 (S)
Naples	*Tehran phlebovirus*	Tehran virus (THEV)	10E2.82	I-47	Ph. papatasi	GQ165522 (L)	GQ165523 (S)
Naples	*Toscana phlebovirus*	TOSV lineage B	10E7.32	MRS2014	*Homo sapiens*	KX010934 (L)	KX010932 (S)
Naples	*Toscana phlebovirus*	TOSV lineage A	10E7.82	189	*Phlebotomus* sp.	KP694240 (L)	KP694242 (S)
Sicilian	*Toros phlebovirus*	Toros virus (TORV)	10E3.42	213	*Phlebotomus* sp.	KP966619 (L)	KP966621 (S)
Naples	*Zerdali phlebovirus*	Zerdali virus (ZERV)	10E6.42	37	*Phlebotomus* sp.	KP966616 (L)	KP966618 (S)

Five previously described NAT protocols incorporating primer sets designed for generic amplification of phleboviruses (“*pan-phlebovirus*” assays) were reviewed and selected (29–33) (Table 2). The primers were reported to amplify partial L and S segments in singleplex (S) and nested (N) formats. The singleplex primer sets S1 and S3 incorporate forward and reverse primer mixes, while the sets S2-3 and N1-2 include a number of wobble bases and inosine in selected positions to increase target coverage (Table 2). The two N2 primer pairs were designed to selectively amplify SFNV and closely related viruses, namely TOSV, Massilia virus (MASV), Punique virus (PUNV), and Zerdali virus (ZERV) (31), whereas S3 was primarily targeting tick-associated phleboviruses (32).

**TABLE 2:** Primer sets used for SBP amplification^*a*^

Assay	Type	Primer sequence (5’−3’)	Target	Position	Product (bp)	Reference
S1	Single round	(F) TTTGCTTATCAAGGATTTGATGC (F) TTTGCTTATCAAGGATTTGACC (R)TCAATCAGTCCAGCAAAGCTGGGATGCATCAT	S segment	129–151 129–150 520–492	370	(29)
S2	Single round	(F) GGCTACTTCAARAAYAARGANGA (R1) CTCTCTCAGICCICCRTGYTG	L segment	2315 2798–2779	505	(33)
S3	Single round	(F) CAGCATGGIGGICTIAGAGAGAT (R) TGIAGIATSCCYTGCATCAT	L segment	2786–2803 3300–3281	514	(32)
S3	Single round	(F) CAGCATGGIGGIYTIAGRGAAATYTATGT (R) GAWGTRWARTGCAGGATICCYTGCATCAT		2786–2814 3309–3281	523	(32)
N1	Nested	(F) ATGGARGGITTTGTIWSICIICC (outer) (R) AARTTRCTIGWIGCYTTIARIGTIGC (outer) (F) WTICCIAAICCIYMSAARATG (inner) (R) TCYTCYTTRTTYTTRARRTARCC (inner)	L segment	2047–2069 2600–2575 2074–2094 2318–2296	553 244	(30)
N2	Nested	(F) CTTYTTRTCYTCYCTRGTGAAGAA (outer) (R) ATGATGAAGAARATGTCAGAGAA (outer) (F) GCRGCCATRTTKGGYTTTTCAAA (inner) (R) CCTGGCAGRGACACYATCAC (inner)	S segment	1143–1169 1583–1561 1183–1205 1505–1486	440 322	(31)

^*a*^ Wobble bases in oligonucleotides are I:Inosine; M:A/C; N:A/T/G/C; R:A/G; Y:C/T; W:G/C.

### *In silico* analysis

**Fig 1:**
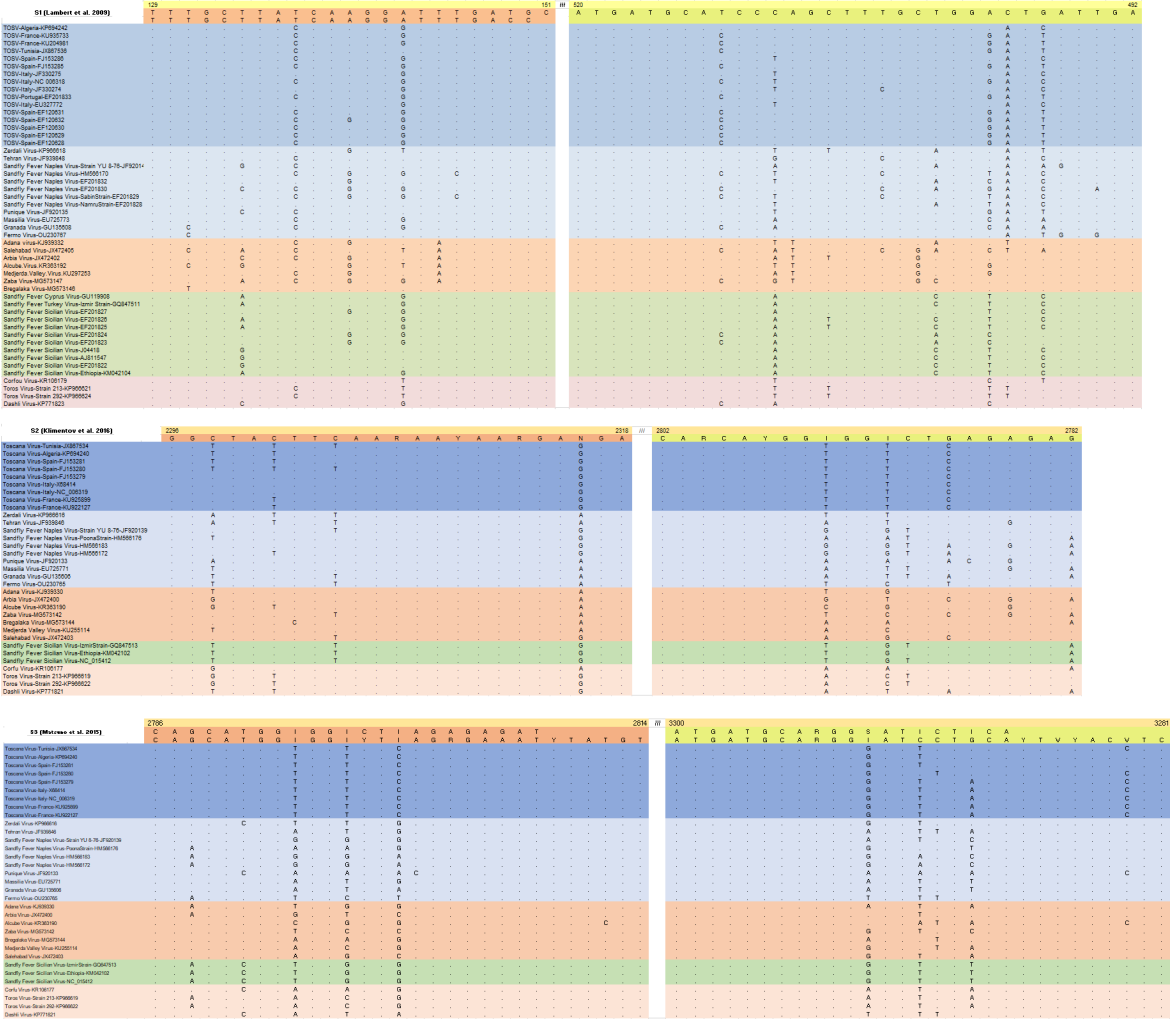
Alignment of the primer sets for singleplex assays. Closely related viruses are indicated in colors (TOSV: dark blue; other SFNVs: light blue; Salehabad group: orange; and Sicilian group: green).

**Fig 2:**
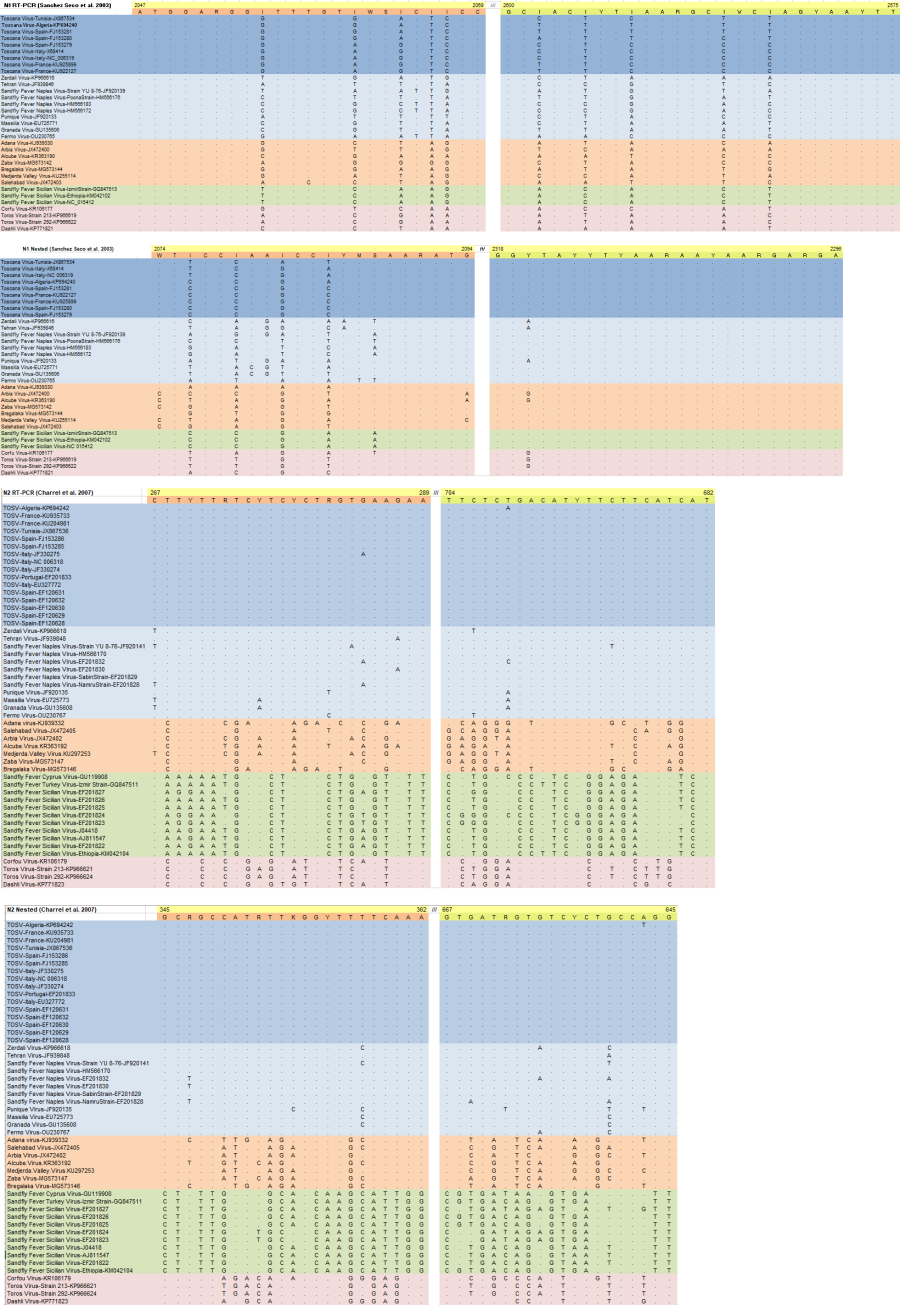
Alignment of the primer sets for nested assays. Closely related viruses are indicated in colors (TOSV: dark blue; other SFNVs: light blue; Salehabad group: orange; and Sicilian group: green).

Primer sequences from all protocols were aligned to the corresponding regions on the SBP nucleocapsid (*N*) and RNA-dependent RNA polymerase (*RdRp*) coding regions, using Clustal Omega (v.1.2.2) (34) (Fig. 1 and 2). The alignments also included viruses closely related to those selected for amplification, as well as sequences from clinical isolates. They were analyzed *in silico* for nucleotide mismatches that might affect primer binding.

The alignments were further used for phylogeny reconstruction to assess virus identification using amplicon sequences produced by the primer sets. MEGA 11 software was used to estimate the optimal substitution model for each alignment and was subsequently employed to infer evolutionary history according to the Akaike information criterion (35).

### Nucleic acid testing and product detection

Nucleic acids were purified using the QIAamp Viral RNA Mini QIAcube Kit according to the manufacturer’s procedures, using the QIAcube system (QIAGEN, Germany). Following purification, the RNA concentration from each strain was optimized at 1.3–3 × 10^1^ ng/µL.

For each primer set, a standardized approach for cDNA synthesis and subsequent amplification was carried out using a unique one-step reverse transcription (RT)-PCR system (Access RT-PCR System, Promega, USA). Each reaction included 1 mM MgSO_4_, 0.2 mM dNTPs, 20 µM of primers, 5 u *Tfl* DNA polymerase, and 5 u AMV RT. Amplifications were performed using a Mastercycler X50s Thermal Cycler (Eppendorf, Germany). The cycling parameters were set as 48°C for 45 min, followed by an initial denaturation at 94°C for 2 min; 40 cycles for denaturation at 94°C for 30 sec; annealing at 55°C for 90 sec; extension at 68°C for 30 sec; and a final extension step of 68°C for 7 min.

The Platinum PCR SuperMix High Fidelity (Invitrogen, USA) was used to do nested amplification using 10 µM of each inner primer from the N1 and N2 sets, using identical thermal cyclers. Platinum PCR SuperMix High Fidelity buffer includes 22 U/mL of complexed recombinant Taq DNA polymerase, *Pyrococcus* species GB-D thermostable polymerase, and Platinum Taq antibody: 66 mM Tris-SO_4_ (pH 8.9), 19.8 mM (NH_4_)_2_SO_4_, 2.4 mM MgSO_4_, 220 µM dNTPs, and stabilizers. The cycling parameters were set as follows: initial denaturation at 94°C for 2 min; 45 cycles for denaturation at 94°C for 15 sec, annealing at 48°C for 30 sec, extension at 68°C for 2 min, and a final extension step of 68°C for 10 min. Products from each primer set were visualized using electrophoresis on 2% agarose gels.

## RESULTS

### *In silico* analysis

Varying numbers of nucleotide mismatches (n: 1–11) were observed in all primer sets (Fig. 1). Inosine and wobble bases in S2, S3, N1, and N2 enabled an extended coverage of diversity in their respective target regions. However, additional positions with mismatching bases were observed: in singleplex pairs, the S1 and S3 primers exhibited the highest and lowest rate of mismatches, respectively, distributed across closely related virus groups. In N1 and N2, an accumulation of mismatches for SFNV and related viruses was noted for N1 inner and outer forward primers (Fig. 2). Likewise, the N2 inner and outer primers were observed to have up to five mismatched nucleotides for SFNV-related viruses, which they were initially designed to target. As expected, N2 demonstrated several mismatches for OWSBPs related to SFSV and Salehabad virus (SALV).

We further performed phylogeny reconstruction using amplicon sequences from each primer set, with additional virus genomes included in the analysis (Fig. 4 and 5). Here, all sets provided sufficient information to resolve the species-based virus taxonomy, as well as discriminate particular pathogen clades (TOSV and SFSV lineages). For the nested sets, products from both rounds demonstrated identical tree topologies, albeit with slightly increased bootstrap support from the first round (Fig. 5).

### Amplification by primer sets

**Fig 3:**
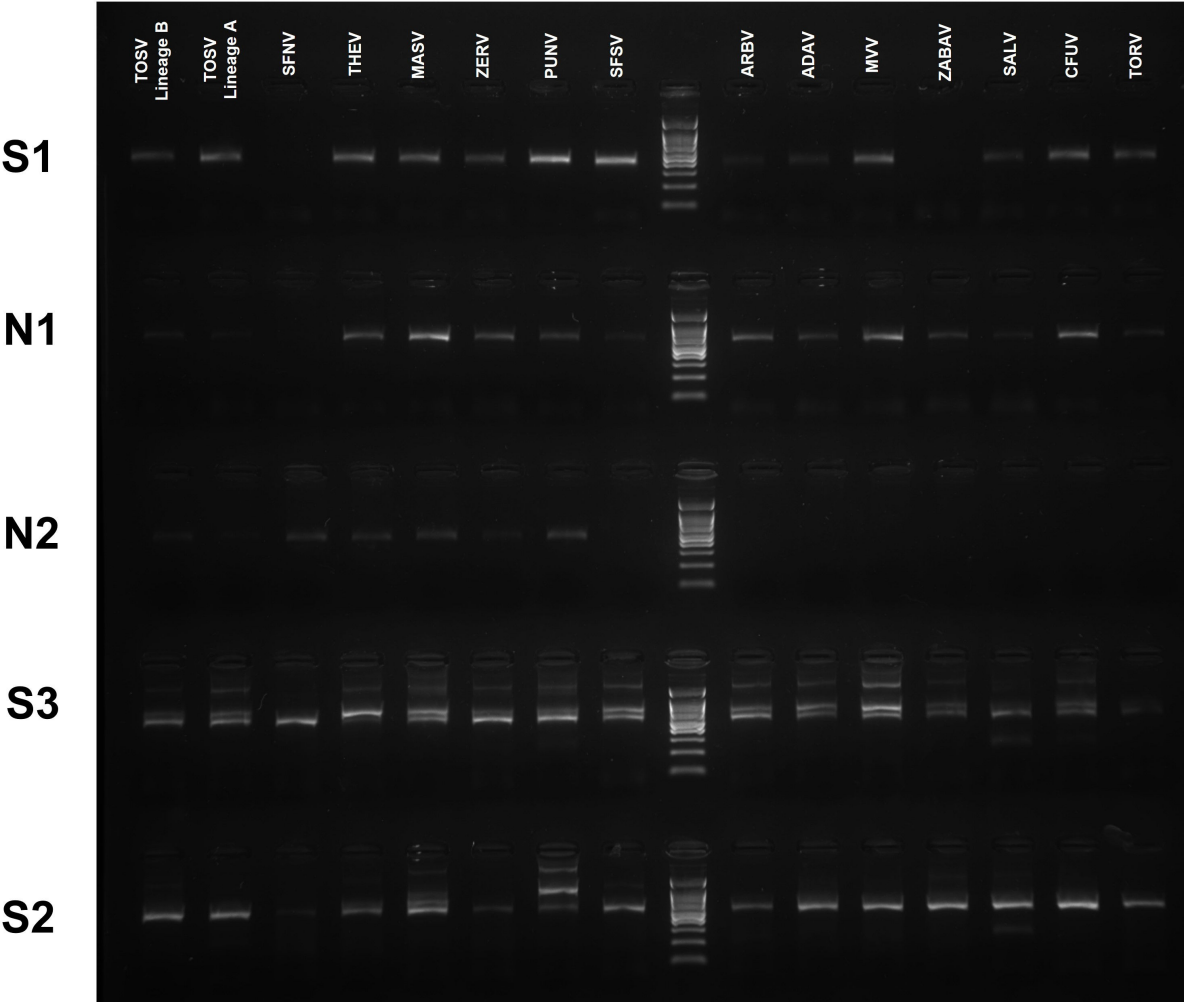
Visualization of the RT-PCR products (S1-370 bp, N1-245 bp, N2-323 bp, S3-524 bp, and S2-507 bp) through agarose gel electrophoresis. Virus strains are indicated using abbreviations.

All virus strains could be detected in at least 80% (4/5) of the sets except for ZABAV, which could be amplified by S2, S3, and N1 exclusively. Using S1, the detection was achieved in 13 (13/15, 86.7%) of the viruses, with SFNV and ZABAV not being detected. The S2 (15/15) and S3 (15/15) sets successfully amplified all virus strains. Incorporating two forward and reverse primers with wobble bases in the reaction mix, the S3 set produced non-specific bands with varying sizes and intensities in several viruses. The expected products from S3 could be visualized as single bands with SFNV and TORV only (Fig. 3). The N1 set could amplify 14 of the viruses tested (14/15, 93.3%), with the exception of SFNV, in parallel with the mismatches observed in the alignments. Designed to amplify selectively seven of the viruses included in the panel (namely MASV, PUNV, SFNV, TEHV, TOSV-A, TOSV-B, and ZERV), N2 achieved totally this objective; interestingly, N2 did not detect any of the eight other viruses, thus demonstrating a good ability to discriminate between viruses belonging to the former Sandfly fever Naples complex and other OWSBPs (Fig. 3). Detection of all known human pathogens (TOSV lineages, SFSV and SFNV) was achieved by S2 and S3, whereas N2 could detect all pathogens within its target range (TOSV and SFNV) (Fig. 3).

Overall, we did not carry out sequencing for amplicons of the expected sizes to confirm specificity because well-characterized and previously titrated virus isolates were used in all experiments. Nevertheless, we selected particular visible non-specific bands for Sanger sequencing, starting from gel-purified material or directly from the amplicons, to rule out the generation of unrelated products in the assays (Fig. 3). These included samples in the S2 set for PUNV and the S3 set for Medjerda Valley virus (MVV). We obtained PUNV sequences from the amplicon and gel extraction products in S2, while in S3, MVV sequences could only be generated from amplicons, presumably due to high nucleic acid loss during gel purification. All sequences showed 100% identity with the target viruses for the same gene region, with larger amplification sizes. They are likely to be generated due to primer attachment at sites in the vicinity other than the original target sites on the virus genome, further facilitated by the standardized conditions for amplification.

## DISCUSSION

Because of the wide distribution of sandflies, large human and animal populations are exposed to sandfly bites and possible infection with at least one and sometimes co-circulating phlebovirus (36). However, the actual public health burden of infections due to SBPs is hard to assess and likely to be underestimated, as the majority of the febrile illnesses due to SPBs do not receive a definitive diagnosis by NAT or serology. The frequently self-limited outcomes of the infections, despite a long and debilitating course resulting in a significant loss of workdays, further impair proper laboratory testing and description of the causative agent (37). Similarly, infections presenting with central nervous system symptoms due to TOSV are likely to be underdiagnosed due to their seasonality, lack of clinical awareness, or availability of appropriate testing (36). Efforts for NAT development and optimization have mostly targeted TOSV- and SFSV-related strains (14–17). The sequence diversity in pathogenic SBPs and naturally occurring strains with unknown pathogenic potential further complicates accurate virus detection by NAT (3). To overcome these questions, PCR detection by generic priming has been widely used for diagnosis and screening. Despite being applicable in detecting known and novel SBPs, these assays have mostly been optimized for in-house testing using well-known virus isolates. Moreover, the technical conditions of each assay are specific and not adapted to parallel screening (29–33). The format of these assays is poorly adapted to clinical diagnosis (due to laboratory contamination risks and lack of standardization); however, they can be utilized as useful tools to understand viral ecology and to select targets that should be prioritized for developing real-time RT-PCR, as it was done for TOSV. Therefore, this study was carried out to adapt previously described generic primer sets to standardized, commercially available reaction components in a one-step RT-PCR format with identical amplification conditions best suited for parallel use, high-throughput formats, and clinical testing. A panel of 15 different virus isolates was used for the evaluation of five primer sets, designed and widely used to detect SBPs (29–33) (Table 2). The viruses included in the panel comprised well-characterized pre-titrate isolates.

Among the primer sets, the S2, S3, and N1 assays target the highly conserved *RdRp* motif on the L RNA segment, whereas the S1 and N2 assays target a conserved region of the nucleocapsid protein encoded by the S segment (Table 2). All sets incorporate multiple primer sets and/or wobble bases to expand the spectrum of virus detection. Initially, we performed a comparative *in silico* analysis of the primer sequences for mismatches and virus strain identification. We observed that they are capable of generating sequence data sufficient for accurate virus description and relevant clade discrimination.

**Fig 4:**
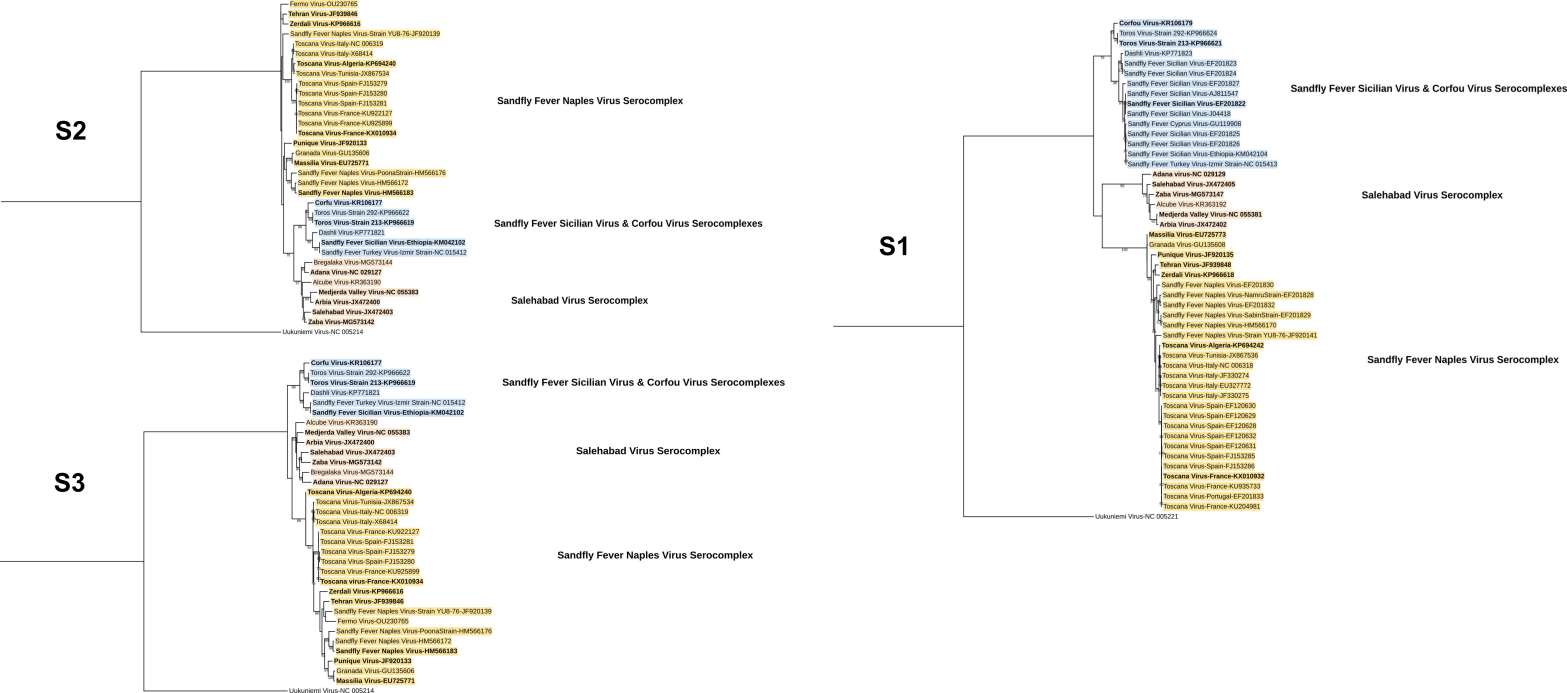
Phylogenetic trees using amplicon sequences of singleplex assays. All trees are constructed using the maximum likelihood method with the general time reversible (GTR) model: gamma distributed with invariant sites (G + I) for 500 replications. Bootstrap values lower than 70 are hidden.

**Fig 5:**
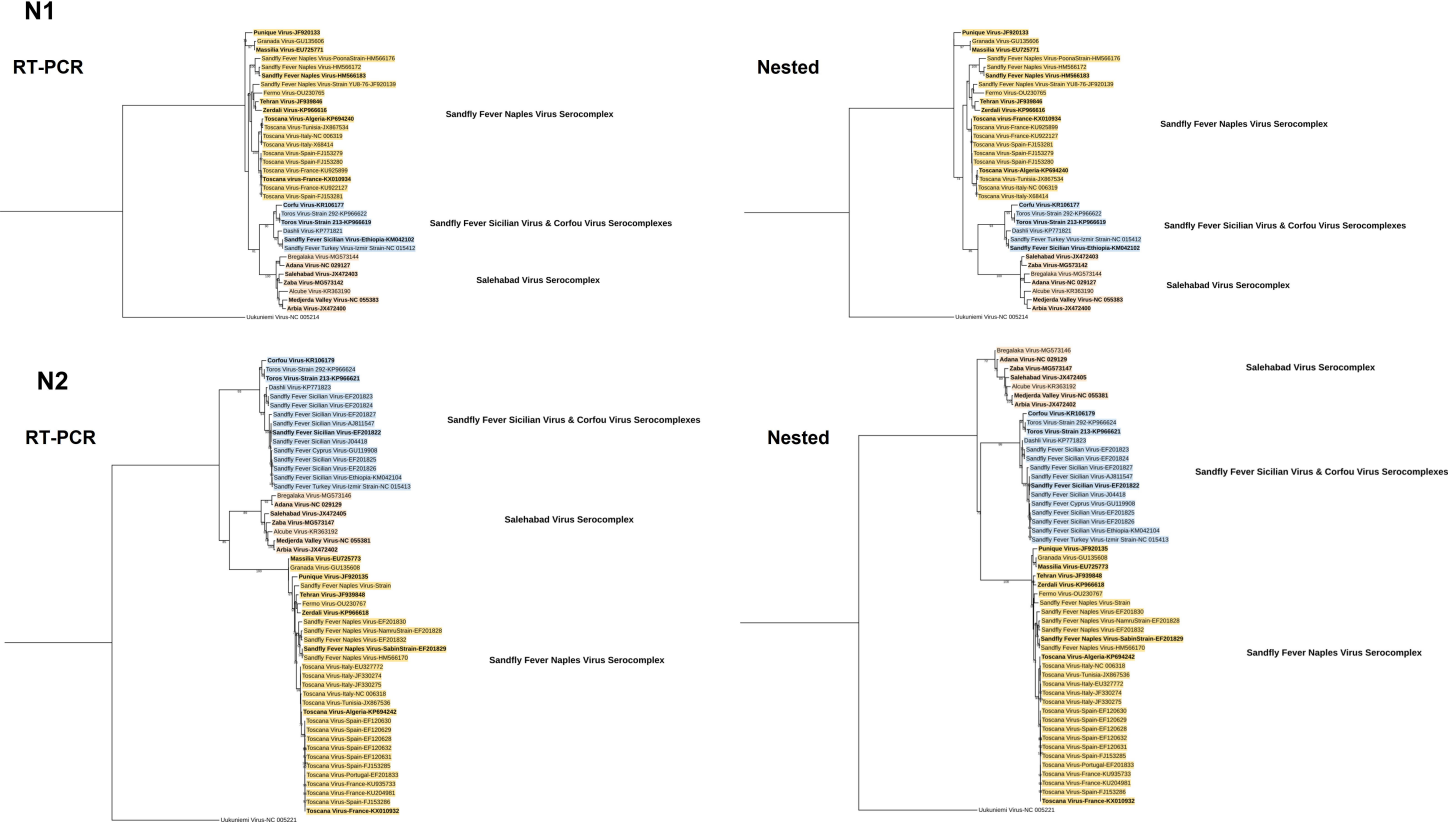
Phylogenetic trees using amplicon sequences of nested assays. All trees are constructed using the maximum likelihood method with the general time reversible (GTR) model: gamma distributed with invariant sites (G + I) for 500 replications. Bootstrap values lower than 70 are hidden.

Using standardized amplification conditions, we observed that S2 and S3 were able to detect all 15 targets (Fig. 3). The few nucleotide mismatches noted in *in silico* analysis had a negligible effect on amplification (Fig. 1). Utilizing several wobble bases to enhance detection range, these sets would be better candidates for developing a generic real-time SBP PCR in a high-throughput amplification setting. However, the non-specific bands observed with the clinically relevant virus strains produced by the S3 set are likely to complicate product detection, particularly in melting curve analysis. It remains to be determined whether this set might benefit from design modifications and primer combinations for improvement. The N1 nested set has been designed to detect all SBPs but was published more than 20 years ago when far less SBPs and their genome sequences were described. The N1 set has been widely used in various settings to detect known SBPs and has been instrumental in identifying probable novel isolates (13, 19, 28). Although it was designed a while ago, it is still accurate and was able to detect all targets except SFNV, despite weak amplification observed with particular virus strains (SALV, SFSV, TORV, TOSV-A, and TOSV-B). It is important to underline that TOSV-specific primers were described in the original study. *In silico* analysis identified a unique mismatch at position 15/21 of the forward nested primer. The N2 nested set was designed in 2007 for the specific detection of viruses belonging to the Sandfly fever Naples serocomplex; in this study, 7/7 target viruses were detected, whereas the 8 targets belonging to other genetic lineages remained negative: this provides support that N2 assay is quite specific and that it could be used in combination with other pan-generic assays for immediate orientation for identification. Second, we analyzed the ability of the sequence of the PCR-amplified genomic region to correctly identify the virus species through either nucleotide alignment or phylogenetic representation (Fig. 4 and 5). For clarity, we indicated virus clustering within the three serocomplexes (Naples, Sicilian, and Salehabad) that have been used for decades within this genus (Fig. 4 and 5).

Due to the utilization of well-characterized virus isolates, we did not proceed to sequence the amplicons of the expected sizes from all virus strains to confirm specificity or identity. However, we selected particular samples with strongly visible, non-specific bands for amplicon sequencing. The findings revealed 100% identity with the target virus and the genome region, suggesting alternate primer attachment to a closer, alternate site to produce larger amplicons. Although accurate phylogenetic assessments should rely on a complete genome sequence, it is obvious that the sequences obtained from these five assays are adequate for virus identification and may facilitate the identification of new viruses, including even the assays producing short amplicons (Fig. 4 and 5).

For both N1 and N2, processing clinical samples should be done carefully due to the risk of carry-over contamination. Specific precautions must be taken, such as the use of a positive control that is known to be absent in the study area and/or the use of a virus that is known to be non-pathogenic for humans to the best of our knowledge (MASV, MVV, and Arbia virus). Nested protocols are also poorly adapted to high-throughput testing and real-time detection. The N1 set has been widely used in various settings to detect known SBPs and has been instrumental in identifying probable novel isolates (13, 19, 28). In this study, despite amplification of the majority of the viruses tested (93.3%), this set failed to amplify SFNV, presumably due to the mismatches observed in the alignments (Fig. 2). These findings exemplify the importance of updating primer sequences as additional virus genomes become available in public databases. The availability and utilization of metagenome sequencing for screening arthropods and environmental samples have accelerated the description of novel viruses and diversity in established taxa (38). Hence, future inspection and update of NAT primers and probes according to the available genome information will be required for not only generic detection approaches but established single-target assays, as well. The accumulation of virus genome data would designate whether sequence updates in established primers would suffice, or new designs would be required for generic SBP detection.

Particular limitations and shortcomings of the strategy we employed should be addressed: we standardized the amplification reaction mix and the total nucleic acid levels for subsequent cDNA synthesis, regardless of the initial titers of individual strains. Repeat testing using identical samples and conditions revealed similar or comparable results (data not shown).

We carried out a comparison of pan-generic systems in the literature using a standardized protocol enabling combination in a single format. We recommend combining at least two systems, depending on the objectives of future studies. These systems are particularly well suited to entomological and virus-discovery studies, and their combination can bring definite benefits alone or associated with metagenomics. The fact that sequencing the PCR products allows species and genetic lineage identification, irrespective of the assay, provides a real added value in virus-discovery studies based on arthropod testing. It should be clear, however, that none of these systems is particularly well suited to routine diagnostics, although they may have a place as research tools for investigating unsolved clinical cases or epidemic situations.

In conclusion, our initial evaluation of published generic primer sets for SBP detection identified two singleplex primer sets with the potential for adaptation in a real-time or high-throughput detection setting. Studies are ongoing to develop and further optimize a preliminary assay and test various hosts and vectors to assess their capacity to detect known and novel viruses.
